# Assessing the impact of waste co-incineration at the Anhovo cement plant (Slovenia) on the regional cancer burden

**DOI:** 10.2478/raon-2025-0050

**Published:** 2025-09-05

**Authors:** Vesna Zadnik, Mojca Birk, Teja Oblak, Maja Jurtela, Sonja Tomsic, Katarina Lokar, Ana Mihor, Nika Bric, Miran Mlakar, Amela Duratovic Konjevic, Tina Zagar

**Affiliations:** Slovenian Cancer Registry, Institute of Oncology Ljubljana, Ljubljana, Slovenia

**Keywords:** cancer incidence, environmental exposure, small area geographical analysis

## Abstract

**Background:**

This epidemiological study aims to assess the cancer risk potentially associated with environmental exposure resulting from cement production and waste co-incineration at the Anhovo cement plant in Western Slovenia and to develop a strong and reliable methodological framework for the long-term surveillance of environmentally related cancer risks in small geographical areas.

**Materials and methods:**

We integrated all the available data sources: cancer cases from the population-based Slovenian Cancer Registry; background population; and available measurements on exposure to air PM_10_ particles and chromium (Cr) in the soil in the municipality of Kanal and the wider Goriška region. Relative risks of cancer in small geographical areas were estimated using Bayesian hierarchical spatial models and the population attributable fractions of the modelled risk factors were calculated. The point source analysis compared the cancer risk near the cement plant to that in more distant areas.

**Results:**

The analysis did not reveal any excess cancer incidence in the area of the Anhovo cement plant or an association with the PM_10_ particles and Cr in the soil. The incidence of mesothelioma remains high in the region, but stable in the last two decades.

**Conclusions:**

In view of the environmental pollution caused by either historical cement production or the potential impact of current waste co-incineration activities in Kanal, we strongly recommend that a follow-up epidemiological study be carried out in the next 10 to 20 years. The methodological framework established in the present study provides a foundation for the ongoing surveillance of the cancer burden in the region.

## Introduction

The development of cancer is a complex, long-term process that is influenced by a range of protective and risk factors that act over decades. It is usually the result of a combination of different factors – including the type, duration and intensity of exposure, as well as the latency period. However, these factors alone do not fully account for the onset of the disease in a particular individual, as genetic predisposition and stochastic (random) events also play a significant role. The European Environmental Agency estimates that around 10% of cancer cases in Europe are due to exposure to environmental pollutants and occupational exposure.^1^

In Slovenia, a Southern European country with a population of 2 million, several epidemiological studies have already been carried out to investigate the association between environmental exposures and cancer risk.^2–5^ One of the hazardous pollutants that increase the risk of cancer in Slovenia is asbestos. The average per capita use of asbestos in Slovenia was identified as one of the highest in a comparison of 53 European countries.^6^ In Slovenia, asbestos was most extensively used in the Anhovo cement factory in the Goriška region of Western Slovenia, where almost 90% of all Slovenian asbestos-related products were manufactured in the middle of the 20th century.^7,8^ Since asbestos exposure is the only well-established risk factor for mesothelioma, the population-based Slovenian Cancer Registry systematically monitors both temporal trends and spatial patterns of mesothelioma incidence.^9^ In the Goriška region – particularly in the municipality of Kanal ob Soči (Kanal), where the Anhovo plant is located – the burden of mesothelioma remains substantial even decades after the ban on the use of asbestos in 1996.^5^

Since around 2007, the Anhovo cement plant has transitioned to alternative fuel sources, in particular through the co-incineration of waste materials. The co-incineration process involves burning waste, including hazardous materials, to generate energy for cement production. During incineration and co-incineration, a mixture of toxic substances is produced and released into the atmosphere, depending on a number of factors. Our recent umbrella review of the literature^10^ showed that there is some evidence of an association between cancer risk and exposure to pollutants from the first-generation of incinerators and the first- and second generations of cement plants. Evidence for a possible association between pollutant emissions from the most modern co-incineration plants and cancer risk is still lacking.^10^

This epidemiological study aimed to utilise advanced geostatistical methods to comprehensively assess the cancer risk potentially associated with environmental exposure from cement production and waste co-incineration at the Anhovo cement plant, focusing on the population of the municipality of Kanal and the wider Goriška region. Another key objective was to develop a robust and reproducible methodological framework for the long-term surveillance of environmentally related cancer risks in the affected region.

## Materials and methods

### Cancer, background population and exposure data

An epidemiological study combining geographic and correlational methods was conducted, in which four key data sources were integrated using geographic information system software: (1) cancer cases from the population-based Slovenian Cancer Registry together with a socioeconomic deprivation index; (2) background population data from the Statistical Office of the Republic of Slovenia; and information on exposure to environmental pollutants from (3) the Slovenian Environment Agency (ARSO) and (4) the Biotechnical Faculty of the University of Ljubljana.

From the SCR^9^, we obtained data on cancer cases diagnosed in Slovenia in the 20-year period from 2001 to 2020, along with mesothelioma data up to 2022; the last ten years, 2011–2020, were used for the geographical analysis (for the previous decades, data is not available on the desired level for small-area geographical analyses). For each cancer case, the information on the patient’s sex, cancer type, age and place of residence at the time of diagnosis (at the level of geographical coordinates), as well as the calendar year of diagnosis and the socioeconomic index Slovenian European Deprivation Index (SI-EDI)^11,12^ categorised in five classes (from the most affluent to the most deprived) were used. Based on a systematic review of the association between cancer risk and exposure to co-incineration pollutants^10^, we identified lung cancer (defined as C33–C34 according to ICD-10 classification^13^), non-Hodgkin’s lymphoma (C82–C85) and sarcoma (as defined in the RareCare study^14^ according to ICD-O-3.2 classification^15^) as cancers that should be studied in relation to exposure. Previous studies^5,8^ have reported a high mesothelioma burden in the Anhovo region attributable to asbestos exposure. That’s why we provide an overview of the cancer burden in the study area for all cancers combined and also excluding mesothelioma cases. In the geographical analysis, where our focus is on non-asbestos environmental exposures, we only report site-specific results in all cases other than the point source analysis. Population data by settlements and grid obtained from Statistical Office of the Republic of Slovenia was further disaggregated by sex and 5-year age groups.

The ARSO provided data on the average daily particulate matter that do not exceed 10 μm in diameter (PM_10_) concentrations in the air for the year 2021, mapped on a 1 km × 1 km grid covering the municipalities of Brda, Kanal, Nova Gorica and Tolmin. The data represents the total air pollution influenced by emissions from the co-incineration plant and other local sources (e.g., residential heating or traffic) and pollution transferred by the winds from the nearby Po Valley in Italy. ARSO calculations were based on a combination of direct measurements and atmospheric modelling.^16^ The modelled PM_10_ values barely differ in the various parts of the study area and are below the official limit values. Nevertheless, we have divided the values (minimum value 12.2 μg/m^3^ and maximum 15.4 μg/m^3^ on a 1 km by 1 km grid level) into three (in this case arbitrary) categories for the purpose of statistical analysis: PM10 less than 14.0 μg/m^3^, PM10 between 14.0 μg/m^3^ and 14.5 μg/m^3^ and PM_10_ more than 14.5 μg/m^3^.

The Biotechnical Faculty of the University of Ljubljana provided data on the chromium (Cr) content in the soil at 30 sampling locations near the waste co-incineration plant, sampled in 2023.^17^ For geographical units without measurements, the value of the Cr concentration was assigned based on the average value at the sampling locations closest to the centroid of the spatial unit, which was determined using Euclidean distance. Similar to the PM_10_ analysis, the Cr measurements (minimum value 14 ppm and maximum value 76 ppm) were divided into three categories for statistical purposes: less than 50 ppm, between 50 ppm and 65 ppm, and more than 65 ppm.

In the absence of historical measurements, present-day concentrations of PM_10_ and Cr were employed as approximations of past exposure. For this reason, the results should not be interpreted as a causal relationship between exposure and cancer risk. We have only selected environmental PM_10_ and Cr as sample data sources for analysing the potential hazardous environmental exposure in air and soil to develop a methodological framework for future monitoring in the affected region, although some further possible cancerogenic substances were also available for analysis.

### Geographical analyses

**FIGURE 1.**
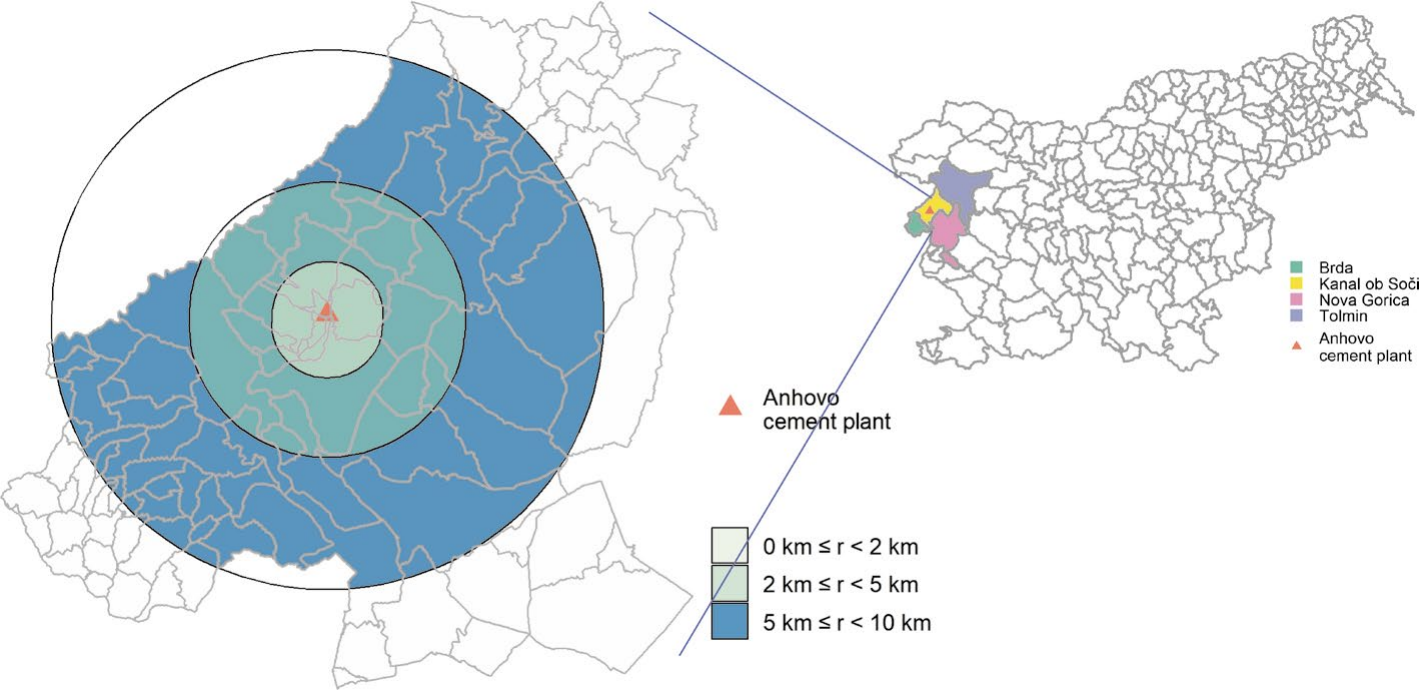
Map of the settlements categorized into three zones constructed for point source analysis and the location of the study area in four municipalities in Western Slovenia. The radius r indicates the distance from the three chimneys of the Anhovo cement plant.

In order to reduce heterogeneity, the smallest possible geographical areas were selected and two levels of geographical subdivision were applied: settlements (the smallest formally defined Slovenian administrative unit) and the 1 km × 1 km grid. The potential excess cancer risk was estimated separately for (a) settlements in four municipalities – Brda, Kanal, Nova Gorica and Tolmin (located in the western part of Slovenia as shown in Figure 1); (b) all the settlements in the municipality of Kanal; and (c) a smaller area within the Kanal municipality (where Cr was sampled). The definitions of the different geographical areas are described in detail elsewhere.^18^

For the point source analysis, a radius map was created with settlements classified into three zones based on the proximity to the three chimneys of the Anhovo cement plant. The first zone extends up to a distance of 2 km from the chimneys, the second zone extends from 2 to 5 km and the third zone from 5 to 10 km from the chimneys (Figure 1). The radii of the zones were chosen to be as small as possible (to detect possible increased cancer risks in the immediate vicinity of the Anhovo cement plant), but at the same time large enough (in terms of population size) to allow relevant estimates to be made. If the pollutants from these point sources (chimneys) have increased the cancer risk, the highest risk would be expected in the first zone, which is closest to the sources, and the lowest in the third zone, which is furthest from the Anhovo cement factory.

The absolute numbers of new cancer cases are strongly influenced by the population size and age distribution in the background population, both of which vary over time and across geographical areas. To account for these differences, we applied a widely used approach to handle unreliable observations in the spatial analyses, namely Bayesian hierarchical modelling. This is the same approach used in our previous studies to estimate the potential excess cancer risk in small geographic areas.^2–4^ An indirect standardisation method was used to calculate the standardised incidence ratios (SIR) by dividing the observed number of cancer cases by the expected number of cases.^19^ The expected number of cancer cases was estimated using Slovenia’s national age-specific incidence rates. SIRs represent an approximation of the relative risk of an individual geographical unit compared to the reference population. A SIR value of 1.0 indicates that the number of cases in the observed population is equal to the expected number of cases in the reference population. SIR values greater than 1.0 indicate more cancer cases than expected and SIR values lower than 1.0 indicate fewer cancer cases than expected.

As cancer is a relatively rare disease, the underlying analyses may be unreliable due to low or no case counts in some small geographical areas. To address this, we used the Besag-York-Mollié (BYM) Bayesian hierarchical spatial model,^20–22^ implemented via integrated nested Laplace approximation (INLA), to smooth the observed values and account for spatial correlation and sampling variability. Spatial clustering was assessed using the ratio of precision parameters (τ_s_/τ_h_). A ratio of less than 1 indicates that the spatial structure accounts for more of the variability than random heterogeneity. To account for potential risk factors (PM_10_, Cr and SI-EDI), we included them as explanatory variables in the BYM model and effectively adjusted the smoothed SIR value for the risk factor. We ran four models: Model 1: without additional explanatory variables; Model 2: SI-EDI variable only; Model 3: one explanatory variable (PM_10_ or Cr); Model 4: two explanatory variables, SI-EDI and PM_10_ or Cr.

The proportion of cancer cases attributable to the risk factor (Population Attributable Fraction; PAF) was calculated taking into account the proportion of the population in each exposure category and SIR same categories.^23,24^

All the analyses were performed using CanMapTool (v1.1)^22^ for cancer incidence mapping, and RStudio (v4.0.2) with the dplyr package (v1.0.2) for data processing. Shapefiles for municipalities and settlements were provided by the Slovenian Surveying and Mapping Authority.

## Results

### Regional cancer incidence with the time trend analyses

Approximately 16,000 people were diagnosed with cancer annually in Slovenia during the five-year period from 2016 to 2020, including 950 (6%) in the Goriška region and just over 50 (0.3%) in the municipality of Kanal. The temporal trends for the ten most common types of cancer in the Goriška region and in the municipality of Kanal do not differ significantly from the trends in Slovenia, with the exception of mesothelioma. Due to the small absolute number of cases in the municipality of Kanal, the 95% confidence interval for the age-standardised cancer incidence rate (ASR) of the individual cancer types is very wide and a comparison of the values for the municipality of Kanal with Slovenia and the Goriška region is not reliable.

**FIGURE 2.**
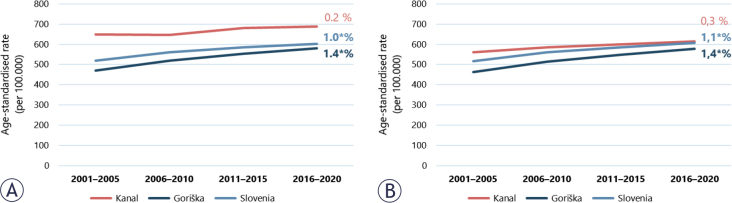
Age-standardised cancer incidence rates (Slovenian standard) with the average annual change (expressed as a percentage) in the Kanal municipality, Goriška statistical region and Slovenia in the 2001-2020 period for **(A)** all cancers and **(B)** all cancers excluding mesothelioma. Statistically significant (at the 5% level) average annual changes are marked with an asterisk.

Similar to the rest of Slovenia, the number of new cancer cases (incidence) in men and women in the Goriška region has been increasing steadily since 1961. The ASR has risen by 16% in Slovenia (an average annual change of 1.0%) and by 23% in the Goriška region (an average annual change of 1.4% in the observed 20-year period) (Figure 2A). In Kanal, the ASR is higher than in Slovenia and Goriška, but remained stable (the average annual change is not statistically significantly different from zero) (Figure 2A). Mesothelioma, which is particularly high in the Kanal municipality, accounts for the largest share of the excess incidence; however, it has not increased in the last two decades. After excluding mesothelioma cases, the ASR for Kanal corresponds to the Slovenian average for the 2016-2020 period and remains stable over the last 20-year period (Figure 2B).

The peak of mesothelioma incidence in Slovenia was already reached in 2004, followed by a steady ASR in the 2004-2014 period and a decline from 2014 to 2022 with an average annual change in the ASR of mesothelioma of -3.1% per year (95% CI is -3.4% to -2.8%). The incidence of mesothelioma in Slovenia is around 40 newly diagnosed cases in the last decade (2013-2022). As expected given the small population in Kanal, the ASR of mesothelioma fluctuates greatly from year to year, but since 1999 the trend of the ASR of mesothelioma in Kanal has remained stable (an average of 6.5 cases per year in the last observed period of 2013-2022). Since 1988, the number of mesothelioma cases in the municipality of Kanal accounts for around 15% of all cases detected in Slovenia and has not changed significantly during this period. However, compared to the incidence of other cancers in Kanal, mesothelioma has decreased from the most common cancer in the 2001–2005 period to the fourth most common cancer in the last ten years. Even today, almost half of all mesothelioma cases in Slovenia are diagnosed in residents of the Goriška region.

### Geographical analysis

#### Analysis by settlements

**FIGURE 3.**
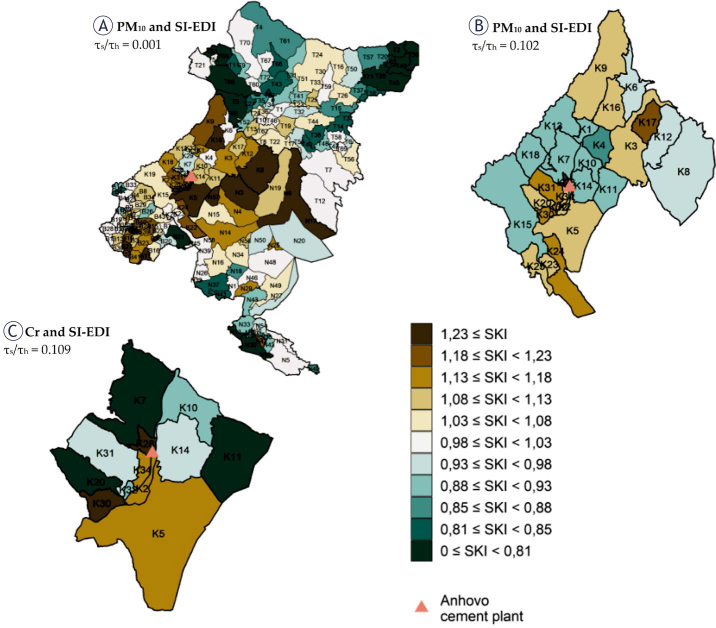
Map of the smoothed standardised incidence ratios (SIRs) of all cancers combined (excluding non-melanoma skin cancer) for the 2011–2020 period: **(A)** for model 4 (with two explanatory variables) with the SI-EDI deprivation index and PM_10_ concentration in the air by settlement in the municipalities of Brda (B), Kanal (K), Nova Gorica (N) and Tolmin (T); **(B)** for model 4 with the SI-EDI and PM_10_ by settlement in the Kanal municipality (K); **(C)** for model 4 with the SI-EDI and chromium (Cr) content in the soil by selected settlements in the municipality of Kanal (K); where Cr measurements were performed. Codes for settlements: B1 = Barbana; B10 = Dobrovo; B11 = Dolnje Cerovo; B12 = Drnovk; B13 = Fojana; B14 = Golo Brdo; B15 = Gonjače; B16 = Gornje Cerovo; B17 = Gradno; B18 = Hlevnik; B19 = Hruševlje; B2 = Belo; B20 = Hum; B21 = Imenje; B22 = Kojsko; B23 = Kozana; B24 = Kozarno; B25 = Kožbana; B26 = Krasno; B27 = Medana; B28 = Neblo; B29 = Nozno; B3 = Biljana; B30 = Plešivo; B31 = Podsabotin; B32 = Pristavo; B33 = Senik; B34 = Slapnik; B35 = Slavče; B36 = Snežatno; B37 = Snežeče; B38 = Šlovrenc; B39 = Šmartno; B4 = Brdice pri Kožbani; B40 = Vedrijan; B41 = Vipolže; B42 = Višnjevik; B43 = Vrhovlje pri Kojskem; B44 = Vrhovlje pri Kožbani; B45 = Zali Breg; B5 = Brdice pri Neblem; B6 = Breg pri Golem Brdu; B7 = Brestje; B8 = Brezovk; B9 = Ceglo; K1 = Ajba; K10 = Kanal; K11 = Kanalski Vrh; K12 = Levpa; K13 = Lig; K14 = Morsko; K15 = Plave; K16 = Ročinj; K17 = Seniški Breg; K18 = Ukanje; K19 = Zapotok; K2 = Anhovo; K20 = Goljevica; K21 = Kamenca nad Ložicami; K22 = Zagomila; K23 = Zagora; K24 = Paljevo; K25 = Prilesje pri Plavah; K26 = Ravna; K27 = Jesen; K28 = Krstenica; K29 = Čolnica; K3 = Avče; K30 = Ložice; K31 = Močila; K32 = Robidni Breg; K33 = Gorenje Nekovo; K34 = Gorenje Polje; K35 = Dolenje Nekovo; K4 = Bodrež; K5 = Deskle; K6 = Doblar; K7 = Gorenja vas; K8 = Kal nad Kanalom; K9 = Kambreško; N1 = Ajševica; N11 = Dornberk; N12 = Draga; N13 = Gradišče nad Prvačino; N14 = Grgar; N15 = Grgarske Ravne; N16 = Kromberk; N17 = Lazna; N18 = Loke; N19 = Lokovec; N20 = Lokve; N25 = Nemci; N26 = Nova Gorica; N27 = Osek; N29 = Ozeljan; N3 = Banjšice; N30 = Potok pri Dornberku; N31 = Preserje; N32 = Pristava; N33 = Prvačina; N34 = Ravnica; N37 = Rožna Dolina; N38 = Saksid; N39 = Solkan; N4 = Bate; N40 = Spodnja Branica; N41 = Stara Gora; N42 = Steske; N43 = Šempas; N45 = Šmaver; N46 = Šmihel; N47 = Tabor; N48 = Trnovo; N49 = Vitovlje; N5 = Branik; N50 = Voglarji; N54 = Zalošče; N56 = Podgozd; N57 = Dragovica; N58 = Sveta Gora; N59 = Pedrovo; N6 = Brdo; N7 = Budihni; N9 = Čepovan; T1 = Bača pri Modreju; T10 = Drobočnik; T11 = Gabrje; T12 = Gorenja Trebuša; T13 = Gorenji Log; T14 = Gorski Vrh; T15 = Grahovo ob Bači; T16 = Grant; T17 = Grudnica; T18 = Hudajužna; T19 = Idrija pri Bači; T2 = Bača pri Podbrdu; T20 = Kal; T21 = Kamno; T22 = Kanalski Lom; T23 = Klavže; T24 = Kneške Ravne; T25 = Kneža; T26 = Koritnica; T27 = Kozaršče; T28 = Kozmerice; T29 = Kuk; T3 = Bukovski Vrh; T30 = Lisec; T31 = Ljubinj; T32 = Logaršče; T33 = Loje; T34 = Modrej; T35 = Modrejce; T36 = Most na Soči; T37 = Obloke; T38 = Pečine; T39 = Petrovo Brdo; T4 = Čadrg; T40 = Podbrdo; T41 = Podmelec; T42 = Polje; T43 = Poljubinj; T44 = Ponikve; T45 = Porezen; T46 = Postaja; T47 = Prapetno; T48 = Prapetno Brdo; T49 = Roče; T5 = Čiginj; T50 = Rut; T51 = Sela nad Podmelcem; T52 = Sela pri Volčah; T53 = Selce; T54 = Selišče; T55 = Slap ob Idrijci; T56 = Stopnik; T57 = Stržišče; T58 = Šentviška Gora; T59 = Temljine; T6 = Daber; T60 = Tolmin; T61 = Tolminske Ravne; T62 = Tolminski Lom; T63 = Trtnik; T64 = Volarje; T65 = Volčanski Ruti; T66 = Volče; T67 = Zadlaz-Čadrg; T68 = Zadlaz-Žabče; T69 = Zakraj; T7 = Dolenja Trebuša; T70 = Zatolmin; T71 = Znojile; T72 = Žabče; T8 = Dolgi Laz; T9 = Dolje

In the settlement-level analysis across the four municipalities for all cancer types combined (2011–2020; Figure 3A), Deskle (code K5 in Figure 3) showed a relative cancer risk approximately 30% higher than the average for the combined municipalities. This finding remained stable after adjusting for covariates including PM_10_, SI-EDI or both. The three models in which we included the explanatory variables SI-EDI and PM_10_ show that the relative cancer risk in the Ložice (code K30) settlement is also over 30% higher. It is also higher in Kal nad Kanalom (code K8) (explanatory variable SI-EDI) and Banjšice (code N3) (model with the joint effect of the explanatory variables PM_10_ concentration and SI-EDI). 3.6% of all cancer cases are attributed to the differences in the socio-economic deprivation index and 3.2% of cancer cases to differences in the modelled values of PM_10_. All the spatial analyses were repeated for selected cancer types (lung cancer, non-Hodgkin’s lymphoma and sarcoma), which did not show an increased relative cancer risk in any of the spatial units examined (maps not shown).

To investigate the impact of the Anhovo cement plant on the surrounding area, we repeated the spatial analysis in a smaller area, only covering the settlements of the municipality of Kanal (Figure 3B) – this analysis did not show an increased relative cancer risk in comparison to the municipality of Kanal as a whole for any of the settlements (including Deskle). We attribute 4.4% of all cancer cases to variations in the SI-EDI deprivation index indicator in these settlements, and based on this analysis, we cannot attribute any increased cancer risk to differences in the modelled values of PM_10_ concentration in the air.

Since soil Cr measurements were only conducted in selected settlements within the municipality of Kanal, the spatial analysis of the association between soil Cr levels and cancer risk was limited to a smaller subset of settlements compared to the PM_10_ analysis (Figure 3C). This analysis also found no evidence of an increased relative cancer risk associated with soil Cr content in any of the settlements within the municipality of Kanal. Due to the insufficient number of spatial units included in this analysis, it was not possible to calculate the attributable cancer burden.

#### Analysis by categories of exposure

In the analysis of the settlements in the municipality of Kanal, classified according to the analysed PM_10_ concentration categories, the relative cancer risk was not statistically significantly increased for any of the three PM_10_ concentration categories or for any of the cancers analysed (all cancer types combined, lung cancer, non-Hodgkin’s lymphoma and sarcoma). No significant increase in risk was found for either of the two spatial levels (settlements and 1 km × 1 km grid) (Table 1). One exception is non-Hodgkin’s lymphoma for the highest category of PM_10_ concentration calculated in a 1 km × 1 km grid (Table 1), which is based on a small number of cases (6 cases in the municipality of Kanal). However, an increased risk of lung cancer and non-Hodgkin’s lymphoma was indicated in association with the higher concentration categories of PM_10_. An analysis of the Cr content in the soil was only possible for selected settlements in the municipality of Kanal where Cr measurements had been carried out. The relative risk was not higher for any of the three categories of Cr content in the soil, for any of the investigated cancer types, or for either of the two spatial levels (Table 1).

**TABLE 1.** Standardised incidence ratios (SIRs) with 95% confidence intervals (CIs) by PM_10_ concentration category and by categories of Chromium (Cr) content in soil for the analysed cancer types for the 2011–2020 period in the municipality of Kanal at two spatial levels (by settlements and by 1 km × 1 km grid).

	Settlements				1 km × 1 km grid		
	Category	Incidence	Population	SIR [95% CI]	cidence	Population	SIR [95% CI]
**Lung cancer**	**PM_10_ concentration in the air**						
	**3 (highest)**	12	1,010	1.49 [0.77–2.60]	12	880	1.68 [0.87–2.94]
	**2 (middle)**	16	2,267	0.89 [0.51–1.44]	15	2,362	0.78 [0.44–1.29]
	**1 (lowest)**	15	2,205	0.89 [0.50–1.46]	19	2,535	0.97 [0.58–1.51]
	**Chromium (Cr) content in soil**						
	**3 (highest)**	0	100	/	1	57	2.49 [0.06–13.87]
	**2 (middle)**	15	1,716	1.04 [0.58–1.72]	16	1,778	1.10 [0.63–1.78]
	**1 (lowest)**	13	1,626	1.01 [0.54–1.73]	11	1,710	0.85 [0.42–1.51]
**Non-Hodgkin’s lymphoma**	**PM_10_ concentration in the air**						
	**3 (highest)**	3	1,010	2.62 [0.54–7.64]	4	880	4.18 [1.14–10.71]
	**2 (middle)**	3	2,267	1.21 [0.25–3.52]	2	2,362	0.82 [0.10–2.96]
	**1 (lowest)**	0	2,205	/	0	2,535	/
	**Chromium (Cr) content in soil**						
	**3 (highest)**	1	100	/	0	57	/
	**2 (middle)**	1	1,716	/	2	1,778	/
	**1 (lowest)**	0	1,626	/	0	1,710	/
**Sarcoma**	**PM_10_ concentration in the air**						
	**3 (highest)**	2	1,010	1.32 [0.16–4.78]	1	880	0.80 [0.02–4.48]
	**2 (middle)**	2	2,267	0.59 [0.07–2.13]	2	2,362	0.59 [0.07–2.14]
	**1 (lowest)**	4	2,205	1.20 [0.35–3.31]	5	2,535	1.48 [0.48–3.45]
	**Chromium (Cr) content in soil**						
	**3 (highest)**	0	100	/	0	57	/
	**2 (middle)**	2	1,716	0.63 [0.08–2.28]	2	1,778	0.62 [0.07–2.24]
	**1 (lowest)**	4	1,626	1.50 [0.41–3.85]	4	1,710	1.49 [0.41–3.82]

#### Point source analysis

**FIGURE 4.**
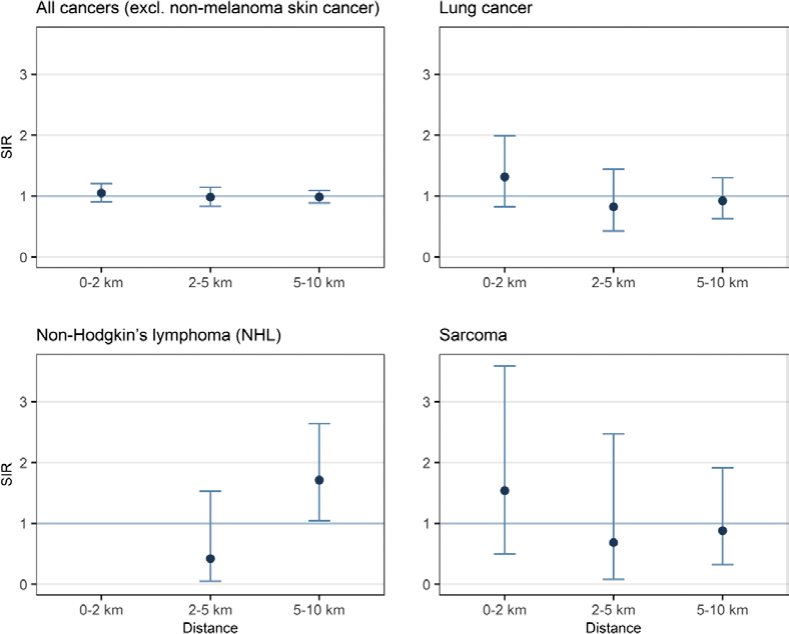
Standardised incidence ratio (SIR) (interpreted as relative risk) with 95% confidence intervals by distance from the three chimneys of the Anhovo cement plant for the analysed cancer types in the 2011–2020 period.

In the settlements of the first zone constructed for the point source analysis (Figure 1), 193 of 2,484 inhabitants developed cancer between 2011 and 2020, of which 22 developed lung cancer, 5 developed sarcoma and none developed non-Hodgkin’s lymphoma. 157 residents developed cancer in the second zone (2–5km) and 379 in the third zone (5–10km). Residents living closer to point sources of pollution (chimneys) did not have a statistically significant increased risk of cancer compared to residents living further away from the chimneys of the Anhovo cement plant. However, the relative cancer risk (SIR in Figure 4) less than 2 km from the chimney is higher for all types of cancer together as well as for lung cancer and sarcomas (Figure 4). There are no residents with newly diagnosed non-Hodgkin’s lymphoma in the first zone (0–2km) in the 2011–2020 period, while SIR is statistically significant for the third zone (1.71, 95% CI is 1.04–2.64). For all the analysed cancer types (all cancers combined, lung cancer, non-Hodgkin’s lymphoma and sarcoma), the risk in the second zone was lower than in the third, so no clear trend can be observed.

## Discussion

The principal objective of this epidemiological study combining geographic and correlational methods was to utilise advanced geostatistical approaches within an epidemiological framework for a comprehensive assessment of cancer risk potentially associated with environmental exposure from cement production and waste co-incineration at the Anhovo cement plant. Our small-area geographical analysis focused on the population of the municipality of Kanal and the wider Goriška region. An additional key objective was to develop a robust and reproducible methodological framework for the long-term surveillance of cancer risk associated with environmental exposures in the studied region.

After adjusting for age, the age-standardised cancer incidence rate in the Goriška region was found to be slightly higher (1.4%) than in Slovenia overall (1.0%). In the municipality of Kanal, the age-standardised cancer incidence rate remained stable over time. Monitoring these trends in the future is important.

Mesothelioma still presents a significant part of the overall cancer burden in the Goriška region, though it is generally classified as a rare cancer type (in Slovenia, around 40 cases per year on average) and presents a small share of the all-cancer burden in Slovenia.^5,9^ During the 2018-2022 period, age-standardised mesothelioma incidence rates (direct standardisation using the World standard) was 0.8 per 100,000 in Slovenia, while it was significantly higher in the municipality of Kanal being at 43.3 per 100,000 population.

The results of this analysis up to the last incidence year of 2022 confirmed our previous findings^9^ that the mesothelioma incidence in Slovenia and in the Goriška region has already reached its peak in year 2004, following the asbestos ban in 1998. After a plateau, the ASR of mesothelioma has been slowly declining from 2014 to 2022 at an average rate of 3.1% per year. Still, the decline in mesothelioma incidence in Slovenia has been slower compared to the decline in import of pure asbestos to Slovenia.^7^ We assume that the exposure of certain Slovenian population groups has continued due to the inappropriate handling of asbestos-containing materials in construction, the unsafe removal of asbestos-containing materials from older residential buildings and its disposal at wild landfills.^9,25^

The results of the spatial modelling did not reveal any increase in the relative cancer risk in the settlements inside the Kanal municipality. However, 4.4% of all new cancer cases in these settlements were attributed to differences in the socio-economic deprivation index SI-EDI.^26^ Studies have confirmed that socio-economic deprivation is associated with cancer incidence in various directions^26^ and findings were replicated by studies using SI-EDI for Slovenia^12^. In Kanal municipality, the employment rate and average income is similar to the Slovenian average.^27^ However, it is an area of combined small rural and rural-urban settlements, where the share of elderly population is high with generally lower socio-economic index.

In the Kanal municipality, we could not attribute any increased cancer risk to differences in the modelled values of PM_10_ concentrations in the air at the smallest spatial levels of settlements. Similarly, there was no increase in the relative cancer risk associated with Cr in the soil in Kanal. Additional modelling of PM_10_ concentrations in the air in a 1 km × 1 km grid revealed significant results for non-Hodgkin’s lymphoma associated with the highest category of PM_10_ concentrations. However, the corresponding 95% CI is wide because it is based on a small number of cases (a total of 6 cases in the municipality of Kanal assigned to three categories of PM_10_ concentration), and the point source analysis shows that the inhabitants with non-Hodgkin’s lymphoma lived in the zone furthest (5–10 km) from the Anhovo cement plant (Figures 1 and 4), a result that is also statistically significant. It must also be emphasised that concentrations of air PM_10_ and Cr content in the soil were below the official limit values for air and soil in all the observed areas^16,17^, including all the different European and Slovenian limit values set to either prevent a risk to human health or to guarantee the suitability of soil for agriculture. In the case of non-Hodgkin’s lymphoma in our study, confounding with non-environmental risk factors, such as certain infections are plausible.^28^

The results of our point source analysis showed that the relative cancer risk in the population residing in a radius of 2km from the chimney of the Anhovo cement plant was increased; however, the 95% CI for lung cancer and especially sarcomas were wide and must be interpreted with extreme caution. Moreover, no clear spatial trend indicating a decreasing relative cancer risk with increasing distance from the cement plant was observed. We assume that the higher overall cancer risk and lung cancer risk near the Anhovo cement plant may have been due to a combination of occupational exposure to the cement production in the past, various socio-economic factors, lifestyle factors (particularly smoking) and different sources of environmental pollution, such as industry, traffic and residential heating.^13,29^ A recent umbrella review summarised that there is moderate scientific evidence that the incidence of cancer overall, and specifically lung cancer, was associated with exposure to hexavalent Cr in workers at the first- to second-generation cement plants, as well as and low to moderate evidence that the incidence of soft tissue sarcoma was associated with exposure to dioxins in the population living near first-generation incinerators; the evidence of a potential association between cancer risk and co-incineration is still lacking in the scientific literature.^10^ However, our modelling of Cr concentrations did not yield any association with cancer risk in the population residing in the observed settlements of Kanal, which is not surprising due to the assessment that the main source of this Cr was probably geogenic.^17^ Additionally, our study did not focus on occupational exposure or model environmental dioxins (or other relevant pollutants). Our study enabled a partial quantification of the joint effects of socio-economic deprivation and environmental pollution, considering all the relevant sources of exposure. The analyses revealed no statistically significant increase in cancer incidence in the area of the Anhovo cement plant, with the exception of the already known high risk of mesothelioma. However, mainly due to the relatively long latency period (discussed in the section below), we cannot completely rule out a possible impact of environmental pollution on cancer risk caused by more recent waste co-incineration at the Anhovo cement plant.

On the basis of our study results, we are proposing further regular detailed epidemiological analyses and close monitoring of the spatial-temporal trends of cancer incidence, including mesothelioma, in the population of Kanal and the Goriška region, while all related evidence-based public health interventions should also be put into practice.

### Strengths and limitations

A key strength of the study is the synthesis of four high-resolution, high-quality data sources within a unified methodological framework. Because of its completeness, longstanding operation, rigorous data validation and national representativeness, the population-based Slovenian Cancer Registry is a gold standard source for population-level cancer research that is particularly suitable for studying spatial, temporal and environmental patterns in cancer incidence and outcomes.^30^ Population-level data is crucial to avoid the selection and participation biases that occur in studies based on cases and selected controls.^19^

For this study, data on cancer cases and the background population were available at the level of residential x- and y-coordinates, enabling the flexible and spatially precise definition of the areas to be analysed, which is a major strength of this study. To assess the sensitivity of our spatial unit definitions, separate analyses were conducted at three levels of spatial resolution: settlements (the smallest available administrative units), a 1 km × 1 km grid, and three distance-based zones relative to the three chimneys of the Anhovo cement plant. The results indicate that the choice of spatial aggregation does not have a meaningful impact on the study outcomes, as the results obtained with different aggregations are consistent. One exception is non-Hodgkin’s lymphoma for the highest category of PM_10_ concentration, calculated using a 1 km × 1 km grid (Table 1), which could also be a consequence of the multiple testing that such an approach entails. In spatial epidemiology, analyses are frequently constrained to the use of administrative units (e.g. regions or municipalities) as the spatial resolution of analysis, due to the limited availability of geocoded data on the individual level. The main problem with administrative areas is that they are arbitrary when mapping the cancer burden, as environmental or behavioural risk factors are usually not aligned with such administrative boundaries.

Permanent residence at the time of cancer diagnosis served as a surrogate measure of exposure status in geographical analyses. People do not necessarily live where they have a registered permanent residence, which also holds for Slovenia.^12^ Further, using a single time-point for exposure determination does not allow for analysing the actual duration and level of exposure in an individual, especially in the adult population, which is generally mobile and may work and spend many hours away from the permanent place of residence, being exposed to other pollutants (including occupational) that are not connected to the area observed in our study. If we take into account the patient’s permanent residence at the time of diagnosis in the analyses, we assume that the patient spent the entire observation period in the same environment, i.e. at the same exposure level (the Slovenian Cancer Registry and Statistical Office of the Republic of Slovenia cannot provide data on residential history). If the patient relocated prior to diagnosis, the impact of risk factors could undoubtedly vary.^31^ Precise data on population migration is not routinely documented in cancer registers, rendering such an analysis unfeasible with routinely collected data.

Important inputs in our study also include recent measurements of air and soil pollutants provided by the ARSO and Biotechnical Faculty of the University of Ljubljana, which are recognised as quality and internationally validated institution and scientific partners. The selection of the two pollutants (PM_10_ particles in the air and Cr in the soil) was partially based on a review of potential carcinogenic substances associated with industrial sources^10^ and partially on a practical need to test the complex methodological framework, while considering the type of available measurements and the time frame of actual exposure measurements. The actual measured concentrations were below the official limit levels for air and soil pollution. Additionally, we did not have at our disposal any information on the form of the Cr. The predominant source of the measured Cr concentrations in the soil in the observed area (i.e. the data we used in the analyses) was assessed to be geogenic^14^, while only hexavalent Cr is carcinogenic.^32^ The range of pollutant concentrations across spatial units was also limited, indicating low variability in exposure levels. Still, our study extrapolates the current PM_10_ and soil Cr exposure levels to represent past conditions, although actual historical concentrations may have varied.

Data on potential confounding factors were limited in this study. Among them, information on smoking habits is particularly important when assessing lung cancer risk. National surveys conducted in the Goriška statistical region^33^ indicated that the smoking prevalence was comparable to or lower than in other regions of Slovenia; however, more granular data at the local level is not available. In our analysis, the Slovenian socioeconomic deprivation index (SI-EDI) was used as a proxy for smoking, based on established evidence that tobacco use is more common in socioeconomically disadvantaged populations.^27^ Other potential confounders include non-industrial sources of environmental pollution, such as traffic emissions and residential heating (e.g. wood-burning stoves). Adjustments for non-industrial environmental sources of pollution may be warranted in future studies, as it was shown that the main sources of PM_10_ air pollution in Kanal and the Goriška region are domestic wood-burning and traffic.^16^

The latency period must be considered in studies on cancer incidence, which has already been discussed previously to some extent. For many cancer types, long-term exposure to risk factors is required for their development, usually spanning between 15 and 20 years for solid tumours^34^ and up to 40 years for mesothelioma.^35^ The population diagnosed with cancer during the observation period (2011–2020) was likely exposed to relevant risk factors around the year 2000 or earlier, including the 1990s – a period when the co-incineration of waste had not yet commenced in the Anhovo cement plant.^36^ Thus, the potential impact of waste co-incineration on cancer incidence could not have been properly assessed yet. For considering the latency period of the carcinogenicity of environmental pollutants related to novel cement production and waste co-incineration, it is necessary to repeat the present study 10–15 years from now.

Small spatial units contain small populations and, consequently, a small number of cancer cases. In particular, the low incidence rates of these events may lead to sparse data in some populations, which means that estimates derived from these models may be unstable and less reliable. A low number of rare events reduces the statistical power of the analysis, making it more difficult to detect significant associations or differences, which can lead to wider confidence intervals and less precise estimates. Aggregating data to larger spatial units was not appropriate for this analysis, as localised exposures could be diluted or masked when results are averaged across heterogeneous regions. For this reason, modern approaches to estimating the relative risk in small spatial areas often rely on smoothing methods^37^, which we also applied in this study. The basic idea behind spatial smoothing is to use information from neighbouring regions to obtain a more stable and less noisy estimate, thus separating the spatial pattern from the noise.^37^ We used the scientifically established and internationally recognised statistical approach of Bayesian hierarchical models, which is robust for small numbers, as it provides more stable estimates and helps to mitigate the impact of rare events.^38^ This approach has been used in our previous studies examining the impact of environmental exposures on cancer incidence in Slovenia.^2–5^

## Conclusions

Considering the presence of environmental pollution, either due to old environmental burdens or to the potential impact of current activities in the municipality of Kanal, it is critical to provide the local population with scientifically robust and unbiased health risk assessments.

In the present study, we developed and applied a comprehensive epidemiologic methodology to assess spatial and temporal trends in cancer incidence and evaluate possible associations with environmental exposures, which has not been done before.

The presented analyses did not reveal any statistically significant excess in cancer incidence in the area of the Anhovo cement plant that could be attributed to environmental pollution. Due to the long latency period between exposure and cancer manifestation – usually 15 to 20 years – the possible effects of current exposure to pollutants are not yet reflected in the cancer figures. However, if the inhabitants of the region were exposed to extreme pollution, an increase in the relative cancer risk could already be observed in the last decade studied, when waste co-incineration was already underway.

The methodological framework developed in this study will serve as a basis for the ongoing monitoring of the cancer burden in the municipality of Kanal and the wider Goriška region. We strongly recommend conducting a follow-up epidemiologic study in the next 10 to 20 years to assess the potential long-term impact of current environmental pollutant exposures on regional cancer incidence, using the present study as a reference. Such a study would also provide an opportunity to re-evaluate the effectiveness of public health interventions aimed at improving the population health in the region. The developed methodological framework allows for the investigation of further types of cancer if future literature provides new evidence related to waste co-incineration. We also recommend that future analyses should include additional measured concentrations of environmental pollutants, along with the latest regulatory thresholds for air and soil quality, to enhance the accuracy of exposure assessment models. In addition, we emphasise that all related evidence-based public health interventions should be actively promoted and easily accessible to the inhabitants.
